# Charting the research frontier for viral infections, immunomodulation, and dementia: a perspective on synaptic biomarkers as essential clinical trial endpoints

**DOI:** 10.3389/fcimb.2025.1701760

**Published:** 2026-01-05

**Authors:** Chun Kiat Lee, Michael Woodward, Mark I Hohenberg, Julian W Tang

**Affiliations:** 1 Molecular & Translational Diagnostics Laboratory, Department of Laboratory Medicine, National University Health System, Singapore, Singapore; 2 Medical and Cognitive Research Unit, University of Melbourne, Austin Health, Heidelberg, VIC, Australia; 3 School of Medicine, Western Sydney University, Penrith, NSW, Australia; 4 Department of Respiratory Sciences, University of Leicester, Leicester, United Kingdom; 5 Clinical Microbiology, University Hospitals of Leicester NHS Trust, Leicester, United Kingdom

**Keywords:** Alzheimer’s disease, CSF synaptic biomarkers, dementia, GAP-43, neurogranin, TDP-43, viral hypothesis, YWHAG : NPTX2 ratio

## Abstract

The viral-inflammatory hypothesis of Alzheimer’s disease offers a new paradigm, yet interventions like antivirals and vaccination present a paradox that challenge therapeutic development. This perspective examines the critical research gap concerning cerebrospinal fluid (CSF) synaptic biomarkers in immunomodulatory therapy trials. Following decades of partially successful amyloid-centric trials, focus has shifted to upstream triggers including viral infections like Herpes Simplex Virus Type 1, Varicella Zoster Virus, and Severe Acute Respiratory Syndrome Coronavirus 2. While large observational and quasi-experimental studies suggest antivirals and vaccines reduce long-term dementia risk, the first major antiviral randomized controlled trial (Valacyclovir for Alzheimer’s Disease) was negative. This perspective posits that this paradox arises from a fundamental flaw in trial design: the absence of synaptic integrity biomarkers. Synaptic loss, not amyloid or tau burden, is the strongest correlate of cognitive decline. Therefore, CSF synaptic protein biomarkers such as the prognostic YWHAG: NPTX2 ratio, postsynaptic Neurogranin (Ng), and presynaptic Growth-Associated Protein 43 (GAP-43) are the most clinically relevant endpoints. The paradoxical trial results may arise from omitting these synaptic measures, creating a mechanistic “black box” obscuring their true biological effects. A strategic framework is proposed, centered on the mandatory inclusion of CSF synaptic biomarkers and relevant co-pathology markers like TAR DNA-Binding Protein 43 (TDP-43; a proteinopathy linked to viral triggers) in all antiviral and vaccine trials. This approach is critical to resolve existing paradoxes, elucidate mechanisms of neuroprotection, and accelerate developing effective therapies that preserve synaptic integrity to prevent and treat dementia.

## 1 Introduction

The landscape of Alzheimer’s disease (AD) research is undergoing a fundamental re-evaluation. Decades of therapeutic development dominated by the amyloid hypothesis have yielded limited clinical success, creating an urgent imperative to explore alternative etiological models (Cummings et al., 2014). In this context, the viral-inflammatory hypothesis has re-emerged as a compelling framework, positing that neurotropic viruses [e.g., Herpes Simplex Virus Type 1 (HSV-1), Varicella Zoster Virus (VZV), and Severe Acute Respiratory Syndrome Coronavirus 2 (SARS-CoV-2)] and subsequent neuroinflammation are critical upstream triggers in sporadic AD (Gannicliffe et al., 1986; Itzhaki et al., 2016). This paradigm is bolstered by evidence reframing amyloid-beta (Aβ) as an antimicrobial peptide (AMP), an innate immune component produced in response to pathogens (Soscia et al., 2010). This model suggests Aβ plaques are a downstream consequence of a chronic immune battle, providing a rationale for why simply clearing them late in the disease has failed.

This renewed focus has generated a compelling, yet paradoxical, body of evidence. Large-scale observational and quasi-experimental studies consistently report that antiviral medications and routine vaccinations are associated with a significant reduction in long-term dementia risk (Eyting et al., 2025; Lopatko Lindman et al., 2021). This optimism, however, is challenged by results from the first major randomized controlled trial (RCT) of an antiviral agent, the Valacyclovir for Alzheimer’s Disease (VALAD) trial, which found no clinical benefit in patients with established mild AD (Devanand et al., 2020; Columbia University Irving Medical Center, 2025).

This perspective posits that the key to resolving this paradox lies not in the interventions themselves, but in the endpoints used to measure their effects. It is unequivocally established that synaptic loss, not the burden of plaques or tangles, is the most proximate biological correlate of cognitive decline in AD (Terry et al., 1991). By focusing on downstream pathologies instead of synaptic integrity, current trial designs have created a mechanistic “black box” that obscures the true biological effects of these therapies. We argue for the mandatory integration of cerebrospinal fluid (CSF) synaptic biomarkers to monitor as well as define both baseline and endpoints in all future immunomodulatory trials to resolve this impasse and accelerate the development of effective, synapse-preserving therapies.

## 2 The re-emergence of the viral-inflammatory hypothesis and the interventional paradox

### 2.1 Re-evaluating the pathogenic cascade

A growing body of evidence implicates several viruses in AD pathogenesis. HSV-1, a ubiquitous neurotropic virus, can reactivate from latency in the trigeminal ganglia and travel to the central nervous system (CNS), where it has been shown to induce Aβ accumulation, tau hyperphosphorylation, and chronic neuroinflammation (Wainberg et al., 2021). This risk is amplified in carriers of the *presenilin* (*PSEN1*) and/or *APOE-ϵ4* allele, representing unmodifiable genetic risk factors for AD (Belbin et al., 2009; Burgos et al., 2002). VZV reactivation (shingles) is also implicated, potentially through a “one-two punch” mechanism where the inflammation from a shingles episode triggers the reactivation of latent HSV-1 already residing in the brain (Cairns et al., 2022). The COVID-19 pandemic further highlighted this link, with SARS-CoV-2 infection associated with long-term neurological symptoms (“long COVID”) and biomarker changes suggesting accelerated neurodegeneration (Gonzalez Aleman et al., 2025).

### 2.2 Aβ as an antimicrobial peptide: reframing a central pathological player

A profound conceptual shift from the pathogen hypothesis is re-evaluating Aβ’s biological role. Compelling evidence supports Aβ functioning not as toxic waste, but as an AMP, a key component of the brain’s innate immune system (Dominy et al., 2019; Soscia et al., 2010; Kumar et al., 2016). In this model, the brain produces Aβ to defend against microbial invasion; the peptide binds to pathogens like HSV-1, forming fibrillar aggregates that entrap and neutralize them. This reframing fundamentally alters the AD pathogenic cascade, suggesting Aβ plaque formation is a downstream consequence of a chronic, costly immune response to CNS infection (Dominy et al., 2019; Soscia et al., 2010; Kumar et al., 2016). In this view, the amyloid plaques are “tombstones” of a long-fought battle between the brain’s innate immune system and the invading pathogens. This provides a rationale for the failure of anti-amyloid therapies. If plaques are a defensive response, removing them late is like clearing a battlefield after the war is lost; it fails to address the underlying conflict (chronic infection and neuroinflammation) driving synaptic loss. Thus, failed amyloid-centric trials may indirectly support the primacy of an upstream infectious trigger. This reordering to an infection/inflammation-first cascade demands a rethinking of therapeutic targets, shifting focus from downstream aggregates to initial immune triggers.

### 2.3 Interventional paradigms and clinical trial paradox

This mechanistic evidence has fueled investigation into antiviral and vaccine interventions, leading to a significant paradox. On one hand, observational data is compellingly positive. Registry studies from Sweden and Taiwan, encompassing hundreds of thousands of individuals, have reported that patients receiving standard antiviral treatment for herpes infections have a significantly lower subsequent risk of dementia compared to untreated individuals (Tzeng et al., 2018; Lopatko Lindman et al., 2021). The evidence for vaccination is even more robust. A landmark quasi-experimental study in Wales, which minimized confounding biases by exploiting a sharp date-of-birth cutoff for vaccine eligibility, found that receiving the shingles vaccine caused an approximately 20% relative reduction in new dementia diagnoses over a seven-year follow-up (Eyting et al., 2025). The consistency of findings across different vaccines suggests a non-pathogen-specific mechanism, such as “trained immunity” where vaccination epigenetically reprograms innate immune cells to promote a more controlled, neuroprotective immune response in the brain (Netea et al., 2016).

In stark contrast, the VALAD trial (NCT03282916), a well-designed Phase II RCT, yielded a definitive null result (Devanand et al., 2020). It enrolled 130 participants with mild AD who were seropositive for HSV-1 or HSV-2 and treated them with high-dose valacyclovir or a placebo for 78 weeks. The trial found no statistically significant difference between the groups in cognitive decline or in the accumulation of amyloid or tau pathology (Columbia University Irving Medical Center, 2025). This discrepancy, however, may be explained by critical design limitations. First, it focused exclusively on treating HSV-1/2 seropositive patients, overlooking the potentially synergistic role of VZV, which is thought to reactivate latent HSV-1 via a “one-two punch” mechanism (Cairns et al., 2022). Second, it was a short (78-week) treatment trial in patients with established disease, whereas the observational data reflects a preventative signal in cognitively normal individuals. By the time AD is diagnosed, the neurodegenerative cascade may be self-sustaining and independent of the initial viral trigger. Finally, a crucial omission in the trial’s design was the absence of any biomarkers for synaptic health, leaving a significant gap in our understanding of the drug’s biological effects.

### 2.4 Causality and therapeutic implications

Distinguishing whether viral reactivation causes AD or results from immunosenescence is critical. The “hit-and-run” hypothesis suggests viruses trigger self-sustaining neuroinflammation that persists independently of replication (Ganz and Ben-Hur, 2024). This implies antiviral monotherapy in symptomatic AD (e.g., VALAD) may fail because the damage is propagated by host immune responses. Consequently, therapeutic strategies must evolve toward dual-targeting: combining antivirals to prevent triggers with immunomodulators to halt downstream synaptic erosion. Validating this link requires synaptic biomarkers; only by observing synaptic preservation during intervention can we confirm the virus drives degeneration.

## 3 The synaptic nexus: a direct measure of cognitive decline

### 3.1 The primacy of synaptic pathology

**Figure 1:**
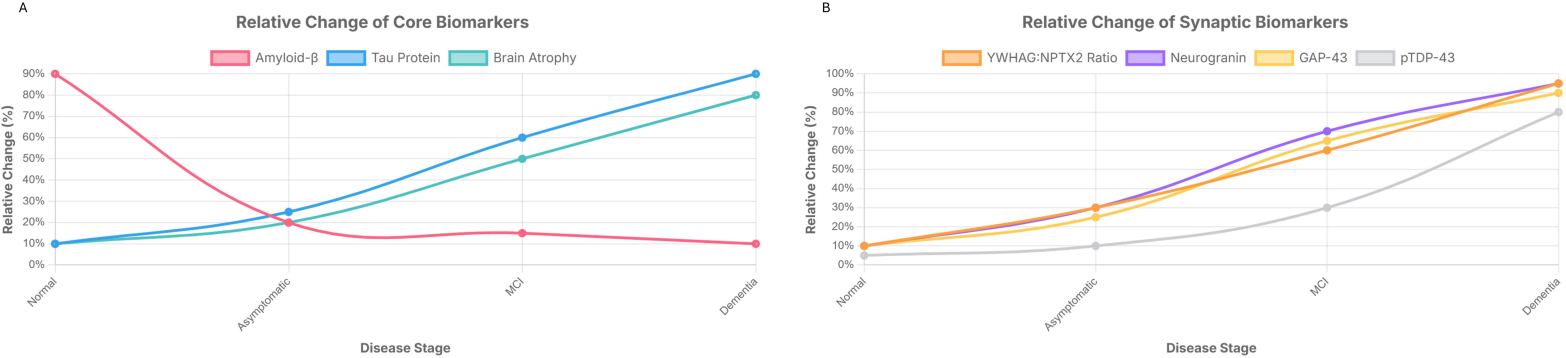
Conceptual progression of Alzheimer’s disease (AD) and synaptic-related CSF biomarkers. The plots represent an idealized progression and assume no additional significant clinical events occurred during this timeline. **(A)** Core AD Biomarkers. This graph illustrates the conceptual progression of the core AD biomarkers, including cerebrospinal fluid (CSF) Amyloid-β (Aβ42), phosphorylated tau (p-tau), and brain atrophy. These biomarkers are shown across the disease continuum from a cognitively normal state to dementia. The curves represent the relative change in their levels over time. **(B)** Synaptic-Related CSF Biomarkers. This graph shows the conceptual progression of synaptic-related CSF biomarkers, including the YWHAG: NPTX2 ratio, Neurogranin (Ng), Growth-Associated Protein 43 (GAP-43), and phosphorylated TAR DNA-Binding Protein 43 (pTDP-43). These biomarkers reflect changes in synaptic function and are plotted over the same disease stages.

While amyloid plaques and neurofibrillary tangles define AD histopathologically (**Figure 1A**; Jack et al., 2018), they are not the strongest biological correlate of clinical symptoms. Foundational research has established that the large-scale loss of synapses is the most robust and consistent predictor of cognitive impairment (DeKosky and Scheff, 1990; Terry et al., 1991) This principle is reinforced by evidence from other neurodegenerative conditions like chronic traumatic encephalopathy (CTE), a tauopathy linked to repetitive head trauma in sports. Modern research in CTE, where cognitive impairment is a key feature, now confirms synaptic damage in athletes after concussion (de Sena Barbosa et al., 2024; McKee et al., 2023). Synaptic dysfunction is an early event, preceding significant neuronal loss and correlating more strongly with dementia severity than the burden of either plaques or tangles (Scheff et al., 2006). Since cognition is fundamentally dependent on the integrity of synaptic networks, synaptic failure is the most proximate biological substrate of cognitive decline. This positions the synapse as the “final common pathway” and the most logical target for measurement in clinical trials.

Recent advances have enabled the quantification of specific synaptic proteins in CSF, providing a direct, *in vivo* window into this core pathophysiology. A panel of these biomarkers offers a multi-faceted view of synaptic health (**Table 1**).

**Table 1:** Profile of key CSF biomarkers.

Biomarker	Biological locus/role	Observed change in alzheimer’s disease CSF	Key clinical correlation/rationale for inclusion
YWHAG: NPTX2 Ratio	Ratio (Regulatory/Postsynaptic Plasticity)	Ratio Increases (YWHAG increases, NPTX2 decreases)	Robustly predicts cognitive decline and conversion to dementia, independently of amyloid-beta and tau pathology. Measures synaptic resilience vs. vulnerability.
Neurogranin (Ng)	Postsynaptic (Dendritic Spines)	Increases	Predicts future cognitive decline, cortical glucose hypometabolism, and hippocampal atrophy. Specific to Alzheimer’s disease postsynaptic injury.
Growth-Associated Protein 43 (GAP-43)	Presynaptic (Axon Terminals)	Increases	Predicts longitudinal cognitive decline and is associated with both amyloid-beta and tau pathology. Specific to Alzheimer’s disease presynaptic injury.
TAR DNA-binding protein 43 (TDP-43)	Nuclear/Cytoplasmic (RNA-binding protein)	Pathological aggregation and mislocalization	Common co-pathology in up to 57% of Alzheimer’s disease cases, associated with worse cognition. Potentially a convergent downstream mediator of viral triggers.

#### 3.1.1 Presynaptic degeneration: growth-associated protein 43

Growth-Associated Protein 43 (GAP-43) is a presynaptic protein essential for axonal growth, synaptic plasticity, and memory formation. In the healthy adult brain, it is highly expressed in the hippocampus and association cortices. In AD, its utility as a biomarker stems from its specific and significant increase in the CSF of patients compared to cognitively normal controls and individuals with other neurodegenerative disorders, including frontotemporal dementia, dementia with Lewy bodies, and Parkinson’s disease (Sandelius et al., 2019; Qiang et al., 2022). This increase is thought to reflect leakage from damaged and degenerating presynaptic terminals into the CSF. Elevated CSF GAP-43 levels show strong correlations with core AD pathologies, including Aβ plaque load and tau tangle burden. Most importantly for clinical trials, high baseline CSF GAP-43 in Aβ-positive individuals is highly predictive of future disease progression, leading to faster brain atrophy, more rapid decline in cerebral glucose metabolism, and steeper cognitive decline. High GAP-43 also significantly increases the risk of converting from mild cognitive impairment (MCI) to AD dementia. Thus, GAP-43 serves as a specific and prognostic marker of presynaptic injury in AD.

#### 3.1.2 Postsynaptic degeneration: neurogranin

Complementing the presynaptic marker GAP-43, Neurogranin (Ng) provides a specific measure of postsynaptic integrity. Neurogranin is a postsynaptic protein concentrated in the dendritic spines of neurons in the hippocampus and cerebral cortex, where it plays a pivotal role in regulating synaptic plasticity and long-term potentiation, the cellular basis of learning and memory. Similar to GAP-43, Ng is depleted from the brain tissue in AD, but its levels become elevated in the CSF, reflecting damage to and degeneration of postsynaptic structures (Portelius et al., 2015; Casaletto et al., 2017). This elevation is a sensitive and early indicator of AD pathology, being detectable even in the predementia, MCI stage of the disease. Higher baseline levels of CSF Ng are a powerful predictor of the rate of future disease progression. In individuals with MCI, elevated Ng is strongly associated with a faster subsequent decline in cognitive scores, an increased rate of cortical glucose hypometabolism as measured by the fluorodeoxyglucose positron emission tomography, and more rapid atrophy of the hippocampus on structural MRI (Portelius et al., 2015). Neurogranin, therefore, provides a robust and prognostic biomarker of ongoing postsynaptic damage, a core feature of the AD process.

#### 3.1.3 Prognostic synaptic dysfunction: the YWHAG: NPTX2 ratio

While GAP-43 and Ng measure the breakdown of existing synaptic structures, a novel biomarker, the ratio of the CSF proteins YWHAG to NPTX2 appears to capture an even earlier state of synaptic dysfunction and stress, providing unprecedented prognostic power. YWHAG is a protein involved in cellular stress responses, while NPTX2 is involved in promoting synaptic plasticity. An increasing ratio likely reflects a fundamental shift from healthy synaptic plasticity to chronic stress and impending degeneration. A landmark large-scale proteomics study identified the CSF YWHAG: NPTX2 ratio as a uniquely powerful biomarker for predicting cognitive outcomes in the AD continuum (Oh et al., 2025). The predictive capacity of this ratio is remarkable. It outperforms traditional biomarkers, including the Aβ42:Aβ40 ratio and p-tau levels, in explaining the variance in cognitive impairment among individuals with AD pathology. In longitudinal analyses, individuals with a high baseline YWHAG: NPTX2 ratio were found to have a 15-fold higher risk of future cognitive decline compared to those with a low ratio. Most strikingly, the study revealed that this ratio begins to increase as early as 30 years before the estimated onset of Aβ plaque and tau tangle formation and 20 years before the manifestation of clinical symptoms (Oh et al., 2025). This makes it the earliest known molecular indicator of AD-related synaptic dysregulation. It is a robust predictor of conversion from cognitive normality to both MCI and dementia, making it an exceptionally valuable tool for identifying at-risk individuals and for measuring disease modification in prevention trials.

#### 3.1.4 A marker of co-pathology: TAR DNA-binding protein 43

The research gap extends to critical co-pathologies that significantly impact clinical outcomes. A prime example is TAR DNA-binding protein 43 (TDP-43) proteinopathy. The mislocalization and aggregation of TDP-43 is not only a frequent co-morbidity in AD but is also the defining pathological hallmark of other major dementias, including Frontotemporal Dementia (FTD) and the more recently characterized Limbic-predominant Age-related TDP-43 Encephalopathy (LATE) (Josephs et al., 2014; Nelson et al., 2019). Viral infections, including SARS-CoV-2, can induce pathological TDP-43 aggregation, suggesting it may be a convergent downstream mediator of virus-induced neurotoxicity (Kim et al., 2025).

## 4 Discussion: a strategic framework for future investigation

The viral-inflammatory hypothesis of AD represents a promising frontier, but its clinical validation has been hampered by a paradox born from a failure of translational methodology. Whilst justified from an end-user perspective to validate the clinical meaningfulness of a therapeutic intervention, the over-reliance on insensitive clinical endpoints and downstream pathological markers like Aβ and tau has obscured our view of the core disease process. To move forward, the field requires a paradigm shift in how therapeutic efficacy is measured. We propose a three-pronged strategic framework.

### 4.1 Redefine trial success with synaptic and co-pathology biomarkers

#### 4.1.1 Define a core “synaptic health panel”

We propose the adoption of a core “Synaptic Health Panel” as a mandatory component of all future immunomodulatory trials. While a specific weighted formula is premature, this panel must provide a multi-dimensional view of the disease. It should include markers for early synaptic dysfunction (YWHAG: NPTX2), presynaptic damage (GAP-43), postsynaptic damage (Ng), and relevant co-pathologies like TDP-43 for comprehensive patient biological pathological stratification (**Figure 1B**).

#### 4.1.2 Use the panel to define trial baseline

This panel, in conjunction with appropriate person-centered cognitive assessments, must be used to define the biological baseline for trial recruitment, ensuring active and control groups start from the same measurable stage of pathology. For prevention studies, the prognostic power of markers like the YWHAG: NPTX2 ratio allows for the enhanced validation of trial cohorts with cognitively normal individuals already on a clear trajectory toward decline. This strategy dramatically increases statistical power, reduces the required trial size and duration, and avoids methodological issues like lead time bias.

#### 4.1.3 Monitor the panel longitudinally

The Synaptic Health Panel, alongside cognitive assessments, must be monitored at regular intervals throughout the trial in both the active and control groups. This longitudinal tracking is essential. By comparing the trajectory of synaptic decline in the treatment group against the natural biological changes observed in the controls, researchers can move beyond simple start- and end-point analysis to definitively assess an intervention’s true effect.

#### 4.1.4 Establish the panel as a key trial endpoint

Finally, we argue for the mandatory integration of this panel to define both baseline and endpoints in all future immunomodulatory trials. The change in the Synaptic Health Panel from baseline should be established as a primary or key secondary endpoint. A statistically significant, positive difference in the biomarker trajectory between the active and control groups would provide the first definitive evidence that an intervention can modify the core pathophysiology of the disease, resolving the current paradox and accelerating the development of effective, synapse-preserving therapies.

### 4.2 From association to mechanism: elucidating molecular pathways

Alongside enhanced clinical trial design, a concerted basic and translational research effort is indispensable to unravel the precise molecular mechanisms at play, particularly the complex interplay between viral triggers, chronic neuroinflammation, and synaptic integrity. We must move beyond simply observing that a virus is associated with AD or that a vaccine is associated with protection, and instead meticulously map the causal pathways from viral infection or vaccine stimulation to changes in synaptic protein expression and co-pathologies like TDP-43 aggregation. This requires advanced human derived models (e.g., iPSC derived neurons and organoids) and *in vivo* models to identify specific, actionable molecular targets.

Translational research should prioritize pathways bridging viral triggers and synaptic failure. First, the Integrated Stress Response (ISR), specifically the Protein Kinase R (PKR) branch, senses viral RNA and halts protein synthesis, depleting synaptic factors like NPTX2 (Costa-Mattioli and Walter, 2020). Second, the cyclic GMP-AMP Synthase - Stimulator of Interferon Genes (cGAS-STING) pathway detects cytosolic DNA, driving microglial-mediated synaptic pruning (Udeochu et al., 2023). Third, the NOD-, LRR- and pyrin domain-containing 3 (NLRP3) inflammasome propagates inflammatory cascades (Heneka et al., 2014). These insights necessitate a strategic pivot to combinatorial interventions. Monotherapeutic antivirals may fail to arrest self-sustaining inflammation. Future trials must prioritize “dual-target” protocols combining antivirals (e.g., valacyclovir) with host-directed neuroprotectants (e.g., ISR inhibitors) to simultaneously neutralize the viral trigger and preserve synaptic resilience, utilizing the Synaptic Health Panel to define therapeutic success.

The goal of this mechanistic research is to identify specific, actionable molecular targets within these pathways. Understanding precisely how a viral infection compromises synaptic structures or how a vaccine-induced immune response bolsters them will provide the foundation for developing novel therapies that can directly target these processes to preserve synaptic and cognitive health.

### 4.3 Building the infrastructure for definitive evidence

The growing dementia crisis makes translating research into public health strategies a global imperative. In Australia, for example, dementia is the leading cause of death for women and is projected to become the leading cause for men (Australian Institute of Health and Welfare, 2024). Generating the necessary large-scale evidence requires investment in a robust and harmonized research infrastructure.

#### 4.3.1 Longitudinal cohorts and global registries

The establishment of large, deeply phenotyped, multi-center longitudinal cohorts with regular collection of biofluids for biomarker analysis, alongside comprehensive clinical, cognitive, imaging, and lifestyle data, is of paramount importance. Such initiatives are already emerging globally, including the Australian Dementia Network (ADNeT) and the UK’s national dementia data initiative, which aim to accelerate research and improve care (Naismith et al., 2023; NHS England Digital, 2024). By tracking synaptic health trajectories in diverse populations with varying histories of infection and vaccination over many years, we can differentiate transient inflammatory changes from sustained effects on synaptic integrity, identify critical windows of vulnerability or resilience, and pinpoint the optimal timing for preventative interventions. Integrating this data into global registries will enable the large-scale, real-world evidence generation needed to detect subtle but population-level effects that smaller trials might miss.

#### 4.3.2 Rigorous biomarker standardization

For these global, multi-center efforts to be meaningful, CSF synaptic biomarker measurements must be rigorously standardized. Significant variability in sample collection, processing, storage, and the specific assay platforms can obscure true biological signals and prevent data comparison across sites. Therefore, a concerted effort, led by international consortia like the Alzheimer’s Association’s Global Biomarker Standardization Consortium (GBSC) and large-scale initiatives like the Alzheimer’s Disease Neuroimaging Initiative (ADNI), is urgently needed (Jack et al., 2018; Ashton et al., 2025). This effort must focus on developing and disseminating unified, best-practice protocols for CSF handling, creating certified reference materials, and establishing external quality control programs specifically for the key synaptic protein biomarkers. Such rigorous standardization is essential to ensure that data generated worldwide is reliable, reproducible, and comparable, forming a solid foundation for therapeutic development.

The call to action is clear. By placing synaptic integrity at the nexus of our investigative efforts, mandating the inclusion of synaptic and relevant co-pathology biomarkers in clinical trials, we can move beyond correlation to establish causation, to de-risk and accelerate drug development, and to finally open the “black box” between intervention and clinical outcome. This synaptic biomarker-driven approach is not merely an incremental improvement in methodology; it is a fundamental and necessary evolution in our strategy to combat virus-associated neurodegeneration. We can resolve the current paradoxes and forge a more direct and promising path toward the discovery of effective interventions to prevent, delay, and treat the neuropathological cascades underlying AD and related dementias.

## Data Availability

The original contributions presented in the study are included in the article/supplementary material. Further inquiries can be directed to the corresponding author.
